# Unicondylar knee replacement versus total knee replacement for the treatment of medial knee osteoarthritis: a systematic review and meta-analysis

**DOI:** 10.1007/s00402-021-03790-7

**Published:** 2021-01-29

**Authors:** Meichao Deng, Yang Hu, Zhongzu Zhang, Hongjun Zhang, Yiming Qu, Gaohai Shao

**Affiliations:** The Department of Orthopedic Surgery, Yongchuan Hospital, Chongqing Medical University, 439 Xuanhua Road, Yongchuan, Chongqing, China

**Keywords:** Meta-analysis, Unicondylar knee replacement, Total knee replacement, Medial knee osteoarthritis, Arthroplasty

## Abstract

**Background:**

Since the optimal surgery for isolated medial knee osteoarthritis (OA) is unclear, this study aimed at comparing the effectiveness of unicondylar knee replacement (UKR) with total knee replacement (TKR) for simple medial knee OA.

**Methods:**

Literature searches of PubMed, Embase, Web of Science, and the Cochrane Library were searched up to 1th April 2020. Only studies comparing UKR with TKR for isolated medial knee OA were included. Data collection and extraction, quality assessment, and data analyses were performed according to the Cochrane standards.

**Results:**

A total of 13 articles with 1888 patients were included, among which, 944 and 944 underwent UKR and TKR, respectively. The analyzed postoperative outcomes were mostly within 5 years of follow-up. The meta-analysis showed that UKR improved knee general function (*P* < 0.00001) and health (*P* = 0.02), moreover, reduced post-operative pain (*P* = 0.01) and complications (*P* < 0.05) more than TKR. There were no significant differences in postoperative revision (*P* = 0.252), high-activity arthroplasty score (HAAS) (*P* = 0.307) and Oxford knee score (OKS) (*P* = 0.15) between the two techniques.

**Conclusions:**

The patients of UKR could achieve better clinical results than that of TKR, moreover, there were negligible differences between the two techniques in postoperative revision in the early and mid-term follow-up and surgeons should be aware of the important reasons for revision of UKR. Thus, UKR instead of TKR should be performed in patients with late-stage isolated medial knee OA.

## Background

Osteoarthritis (OA) is the main cause of disability in elderly individuals, with 50% of lifetime risk of symptomatic knee arthritis [1]. Knee arthroplasty is an effective and routine surgery for the treatment of knee OA [2]. However, it is estimated that up to 47% of patients in need of knee arthroplasty have unicompartmental disease [3]. Because the medial ventricle of the knee changes when moving less than the lateral or patellofemoral joints, OA wear is mainly present in the medial compartment, accounting up to 50% of patients [4]. Unicondylar knee replacement (UKR) and total knee replacement (TKR) are two major surgical options for medial knee OA. In UKR, only the damaged knee compartment is replaced, while, in TKR the total knee compartment is replaced [5].

Since UKR, first performed in 1954, has been developed for more than 50 years, it has evolved from a limited-use operation to an effective single-chamber OA bone preservation operation [6]. However, UKR is performed in a limited surgical volume and is a complex technique with a steep learning curve, only a few arthroplasty surgeons offer this option to their patients [7, 8]. UKR accounts for only 9% of all knee replacements in the UK [3]. On the contrary, TKR is a widely accepted, reliable, cost-effective and suitable surgical method for patients with end-stage OA targeting at pain reduction, function restoration and improvement of health-related life quality [9, 10]. Despite these positive effects, 17–19% of patients were dissatisfied after TKR [11]. Lyons et al. reported that the UKR survival rates were 95% and 90% over 5 and 10 years respectively in a large retrospective database analysis, which are lower than that of TKR [12]. In contrast, a recent report found that 432 medial UKR patients had a survival rate of up to 97.5% over an average follow-up of 5.7 years [7]. There are no comprehensive studies focusing on the optimal choice of surgical methods for medial knee OA and most studies investigating were based on the surgeon's preference [13, 14]. Therefore, TKR and medial UKR are still controversial as treatment options for medial knee OA.

Several meta-analysis [15–17] have compared the therapeutic effects of UKR and TKR on knee arthritis, but the inclusion criteria for patients were not strict enough, and the study populations of the included studies were not uniform. To some extent, these factors affect the performance comparison of the two surgeries on medial knee OA.

The purpose of this study was to conduct a meta-analysis comparing early and mid-term functional outcomes, complications and revision between UKR and TKR for medial knee OA. In this meta-analysis, it is hypothesized that UKR has higher functional accuracy and fewer complications than TKR, but that there are no differences in revision between the 2 techniques.

## Methods

### Literature search and information sources

Two researchers independently searched the Cochrane Library, PubMed, Embase, and Web of Science databases from their inception through April 1, 2020, and manually searched the remaining relevant literature from the references included in articles. In the search process, restrictions were placed on the English language but not on the year of publication. The following primary search terms were used: “unicompartment OR unicondylar OR condylar OR partial,” “total,” “knee,” “arthroplasty OR replacement,” and “medial”.

After the initial database search, references of the relevant articles were searched manually by 2 researchers to identify additional studies.

### Study selection

Research selection is developed and implemented in accordance with the Preferred Reporting Items for Systematic Reviews and Meta-analyses statement (PRISMA) [18]. Studies were selected based on the following inclusion criteria: (1) study design: randomized controlled trial (RCT) or nonrandomized controlled study; (2) patients diagnosed with isolated medial knee OA with functionally intact anterior cruciate ligament which means medial compartment arthritic change exceeds grade II, or complete radiological joint space loss exists in the medial compartment, and the lateral or patella-femoral compartment arthritic change doesn’t exceed grade II; (3) intervention: UKR vs TKR; (4) both operations were not be performed in the same patient; (5) follow-up for at least 6 months; (6) when the same author or author group published multiple research articles on the outcomes of UKR and TKR, we typically included only the most recently published data; and (7) studies included the following outcomes:

Primary outcomes: (1) knee function scores including Bristol knee score (BKS), Knee Society score (KSS), new KSS, Oxford knee score (OKS), and high-activity arthroplasty score (HAAS); and (2) postoperative revision.

Secondary: (1) postoperative health quality: pain score and EuroQol-five dimensions visual analogue score (EQ-5D VAS); and (2) complications: total complications, deep vein thrombosis (DVT), blood transfusion rate and postoperative manipulation under anesthetic (MUA).

The exclusion criteria were as follows: (1) duplication of literature; (2) meta-analyses, systematic reviews, case reports, editorials, letters, abstracts, nonhuman studies and cadaveric experimental studies; (3) studies without usable data; and (4) studies unrelated to this study.

Two researchers independently screened the titles and abstracts of the studies and selected applicable studies for full-text review. The selection of articles was performed based on reviewer consensus. Disagreements over the literature selection were resolved by a third reviewer.

### Assessment of methodological quality

Two reviewers independently evaluated the methodological quality of the included studies. For RCTs, Cochrane risk-of-bias tool was utilized to assess the quality of the study [19]. The following domains were assessed: sequence generation, allocation concealment, blinding of participants and personnel, blinding of outcome assessment, incomplete outcome data and selective reporting. For non-RCTs, the Newcastle–Ottawa quality assessment scale (NOS) was used with a total score of 9 and higher scores represent higher quality [20]. The final decision was based on the consensus of the reviewers, and any differences were resolved by the third reviewer.

### Data extraction

Using a predefined data extraction form, 2 reviewers independently extracted the following data from the selected studies: first author name, publication date, country, number of patients, the average age at surgery, sex, body mass index (BMI), average follow-up time, patient-reported outcomes, complications, and revision rate. The patient-report and complication analyzed in this study were the latest follow-up results for each study. The continuity variables included the sample size, mean value, and standard deviation (e.g., KSS); the dichotomous variables included the sample size of occurrence, sample size without occurrence, and total sample size (e.g., complications). If the data could not be obtained, we tried to contact the corresponding author for details by email at least three times. If the author did not reply to the email or accurate data could not be obtained, the relevant data in the study were excluded from the analysis.

### Statistical analysis

The extracted data were pooled using RevMan 5.3.5 software (Cochrane Collaboration). Odds ratios (ORs) were calculated for the dichotomous variables in each study. Weighted mean differences (WMDs) were calculated for continuous variables, and 95% confidence intervals (CIs) were calculated for all effect sizes. The Higgins *I*^2^ statistic was calculated to test the heterogeneity among different studies: (1) if *I*^2^ ≤ 50%, there was no obvious heterogeneity among the studies, and the fixed effects model was used to pool the data; (2) if *I*^2^ ≥ 50%, the heterogeneity among the studies was considerable, and the random-effects model was used to pool the data [21]. Obvious clinical heterogeneity was treated by performing subgroup analysis or only descriptive analysis. Potential publication bias was assessed by Begg’s and Egger’s tests [22]. Forest plots were used to present the results of the individual studies, and from a statistical perspective, the effect size was eliminated. A *P* value < 0.05 was defined as statistically significant for all tests.

## Results

### Study selection

**Fig. 1:**
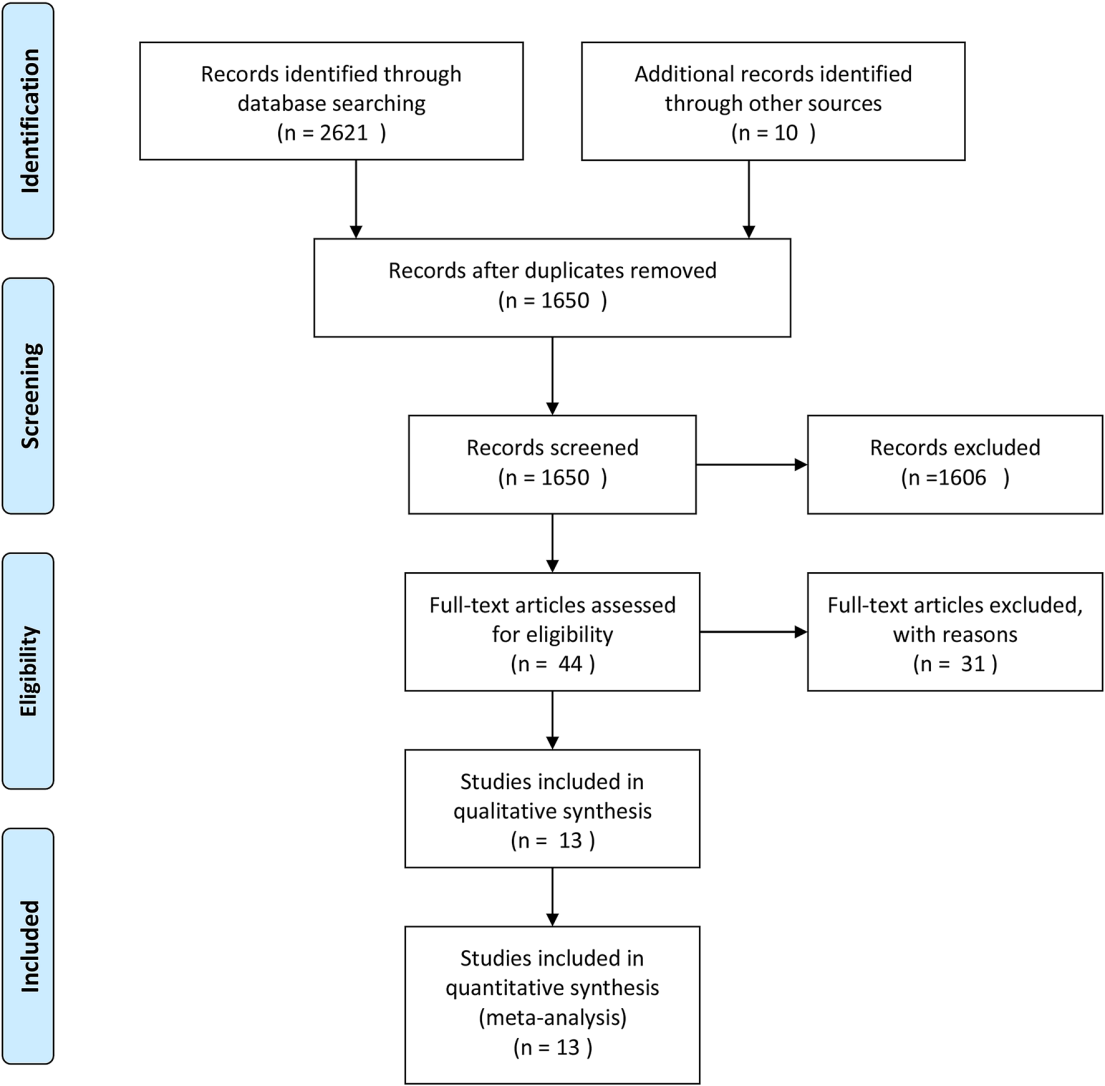
Flow of trials in the meta-analysis

Figure 1 outlines the study selection process. According to the retrieval strategy, 2621 studies were identified through the literature search, including 502 studies from the Cochrane Library, 1320 studies from PubMed, 631 studies from Embase, and 168 studies from the Web of Science. Ten studies were identified via a manual search. After reviewing the studies, we removed duplicate studies and obtained 1650 articles. After screening the titles and abstracts, 44 related studies were first screened out. After conducting a full-text review on the remaining studies, 13 studies were left in the final analysis, including 4 RCTs and 9 non-RCTs (cohort and case–control studies) [3, 5, 9, 12, 13, 23–30].

### Study characteristics and risk-of-bias assessment

The 13 studies included 944 cases of UKR and 944 cases of TKR, of which the follow-up duration was almost within 5 years. However, only one study reported the outcome of 15 years of follow-up, in which the author published a total of 3 articles about the results of different times from the same group of patients [27, 31, 32]. Therefore, the latest results of the relevant data were extracted. The main characteristics of these studies and the patients are summarized in Table 1.

**Table 1:** General characteristics of the included studies

Study	Study design	Country	Follow up (year)	Sample size	Gender (male)	Age	BMI (kg/m^2^)	Results
				UKR/TKR	UKR/TKR	UKR/TKR	UKR/TKR	
N. D. Clement (2020) [23]	Case–control	UK	0.5	30/90	24/68	65.9/67.8	30.5/29.7	(5) (6) (7)
Jason L. Blevins (2020) [12]	Cohort	USA	2	150/150	84/84	62.6/65.2	28.9/29	(1) (3) (6) (9) (12)
David J Beard (2019) [3]	RCT	UK	4.9	264/264	153/153	65.2/64.7	31/31.8	(1) (3) (5) (7) (8) (9) (10) (11) (12)
Georg Hauer (2019) [25]	Case–control	Austria	2.3	35/35	10/13	66/66	28.7/28.5	(3) (6)
Geert Peersman (2019) [5]	Cohort	Belgium	1	57/62	27/20	64/66.5	< 40	(1) (3) (7) (9) (10) (11)
David S. Casper (2019) [26]	Cohort	USA	2	83/50	44/31	64.3/63.1	28.6/28.5	(4)
Suzanne Witjes (2019) [9]	Cohort	Netherlands	2.2	100/68	41/32	63.6/68.7	25.9/29.6	(4) (6)
Jijun Zhao (2019) [24]	Cohort	China	1.9	32/28	12/11	68.6/69.2	–	(6) (9) (10)
Vikas Kulshrestha (2017) [13]	RCT	India	2	36/36	6/10	59.7/62.2	28.3/27.5	(1) (5) (7) (8) (9) (10) (11)
Peng Fei Sun (2012) [28]	RCT	China	4.3	28/28	10/9	60/61	30/30	(1) (3) (9) (10) (11) (12)
A. Manzotti (2007) [29]	Case–control	Italy	3.8	34/34	14/14	69.08/70.7	< 30	(1) (3)
K Y Yang (2003) [30]	Cohort	Singapore	0.5	50/50	8/6	65.1/66.5	–	(9) (10)
John H. Newman (1998) [27]	RCT	UK	15	50/52	17/21	69.6/69.8	–	(1) (2) (6) (9) (10)

(1) Revision; (2) Bristol knee score (BKS); (3) Knee Society score (KSS); (4) New KSS; (5) Oxford knee score (OKS); (6) Pain evaluation; (7) EuroQol-five dimensions three level (EQ-5D-3L) score; (8) High-activity arthroplasty score (HAAS); (9) Total complications; (10) Deep vein thrombosis (DVT); (11) Blood transfusion; (12) Manipulation under anesthetic (MUA); *UKR* unicondylar knee replacement, *TKR* total knee replacement, *RCT* randomized controlled trial, *BMI* body mass index

**Fig. 2:**
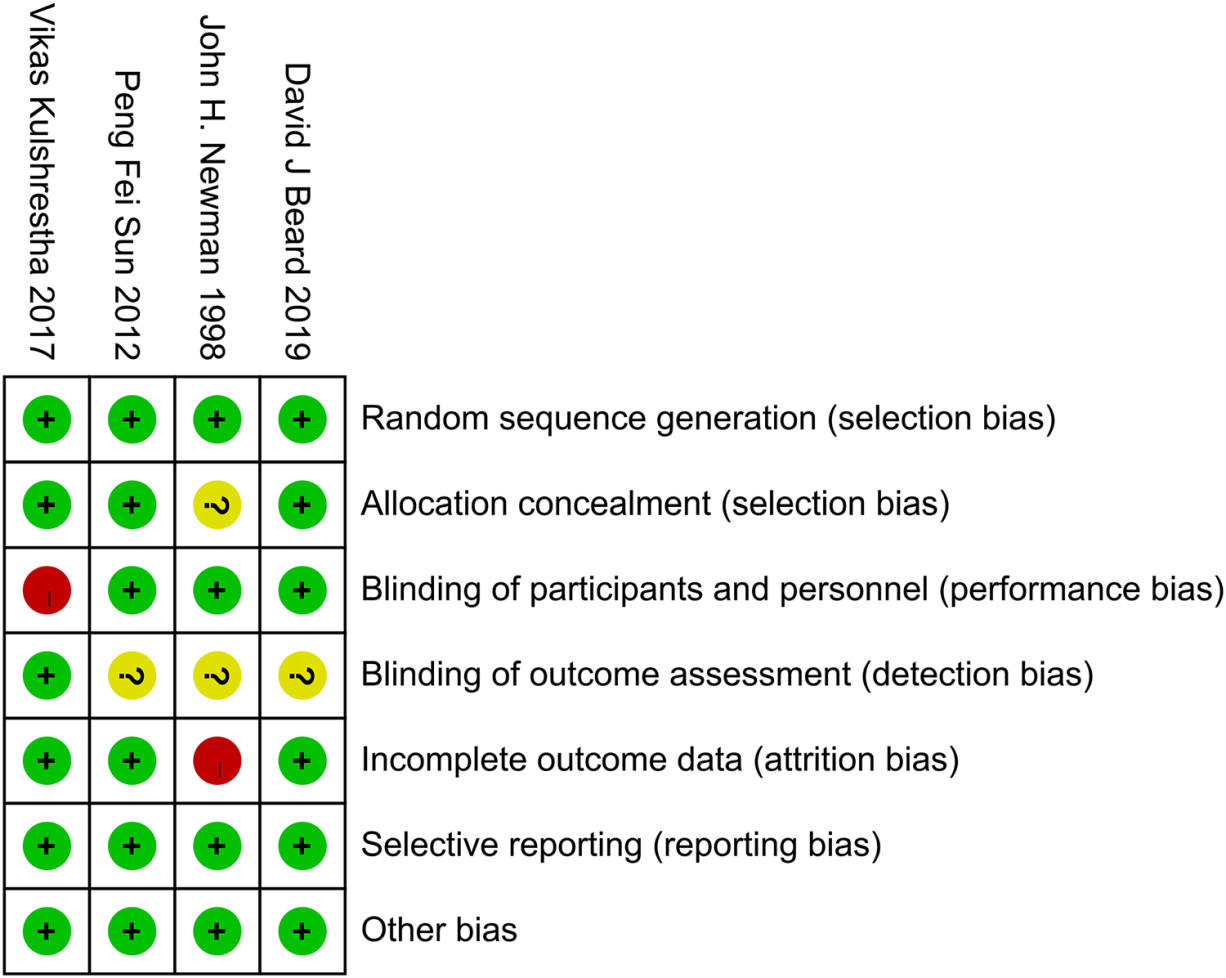
The risk of bias summary for RCTs: green, no bias; red, bias; yellow, unknown bias

**Fig. 3:**
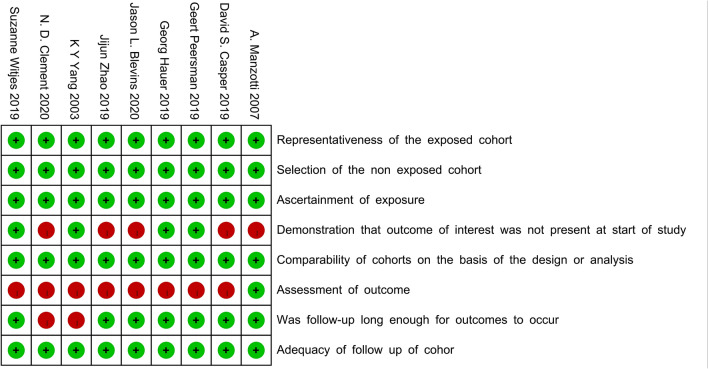
The risk of bias summary for non-RCTs: green, no bias; red, bias

Quality assessment for the included studies was evaluated based on the above-mentioned principles. All RCTs described in detail the inclusion–exclusion criteria, as well as the randomization methods, and only one study did not report allocation concealment schemes. One of the studies noted that the surgeon might make a final decision on the perioperative findings, which probably affected the random principle of the study. Blindness in the evaluation of the results of three studies was not reported, which may increase the test bias. None of the studies showed an unclear bias due to selective outcome reporting or other bias (Fig. 2). The NOS scores for the non-RCTs ranged from 6 to 8, which indicated that all of the included non-RCTs had high quality (Fig. 3).

### Results of the meta-analysis

#### Comparison of the postoperative knee function

**Fig. 4:**
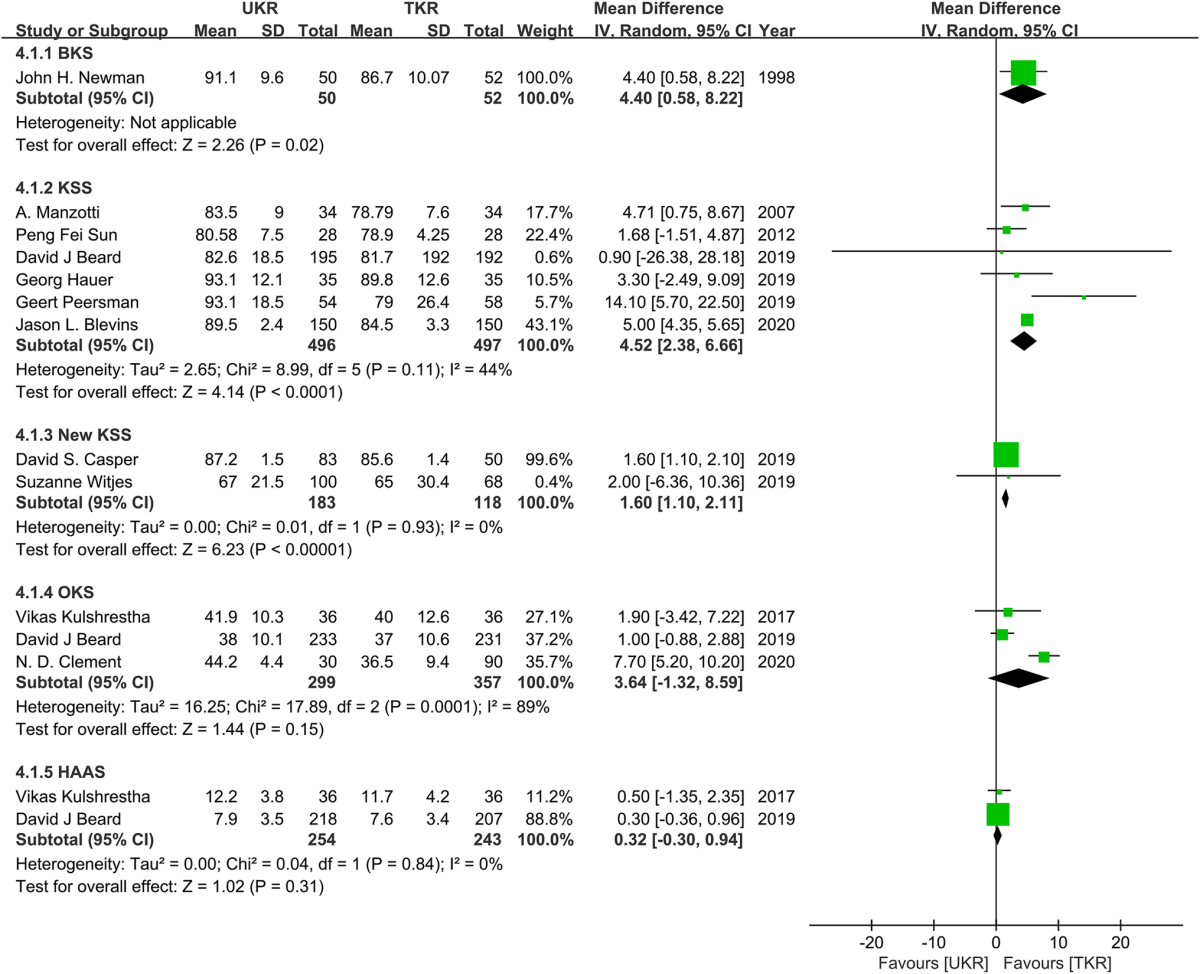
Forest plots of the knee function scores of UKR and TKR

Nine studies involving 729 UKR patients and 667 TKR patients were included in the knee function analysis. The pooled data from 6 studies indicated a significant benefit favoring UKR over TKR in KSS function (WMD = 4.52; 95% CI = 2.38–6.66; *P* < 0.0001; *I*^2^ = 44%). The pooled data from 2 studies demonstrated a significant benefit favoring UKR over TKR in new KSS function (WMD = 1.60; 95% CI = 1.10–2.11; *P* < 0.00001; *I*^2^ = 0%). However, there were no significant differences between UKR and TKR in terms of postoperative OKS (WMD = 3.64; 95% CI = − 1.32–8.59; *P* = 0.15; *I*^2^ = 89%) and HAAS (WMD = 0.32; 95% CI = − 0.03–0.94; *P* = 0.31; *I*^2^ = 0%) (Fig. 4).

#### Comparison of the postoperative revision

**Fig. 5:**
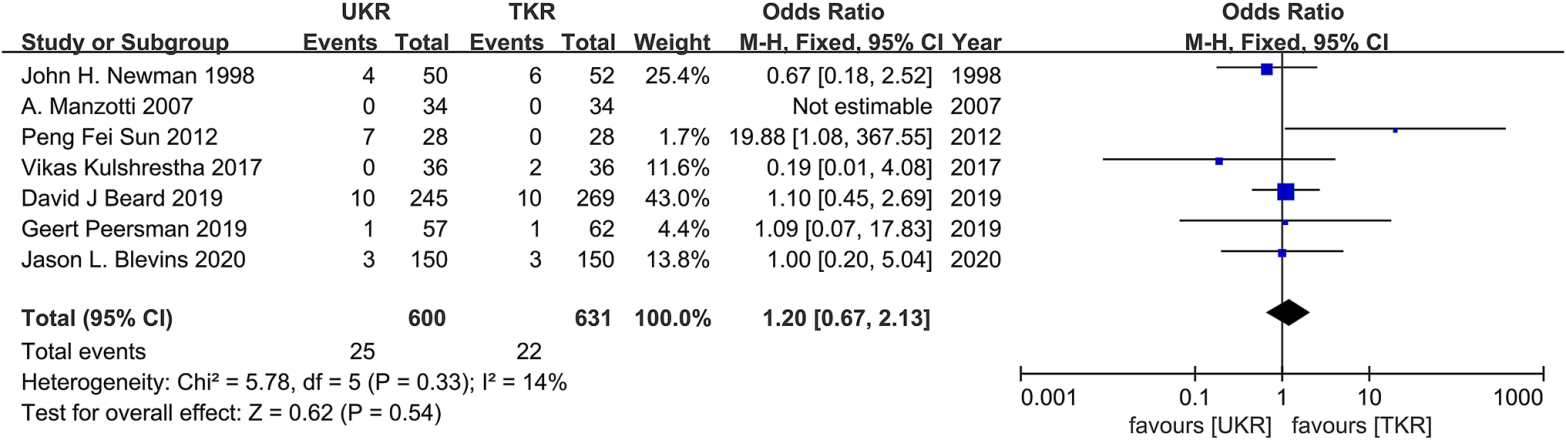
Forest plots of the postoperative revision of UKR and TKR

Seven studies involving 600 UKR patients and 631 TKR patients were included for the postoperative revision analysis. The pooled data revealed no significant differences in revision (OR = 1.20; 95% CI = 0.67–2.13; *P* = 0.54; *I*^2^ = 14%; Fig. 5) between the groups.

#### Comparison of the postoperative health quality

**Fig. 6:**
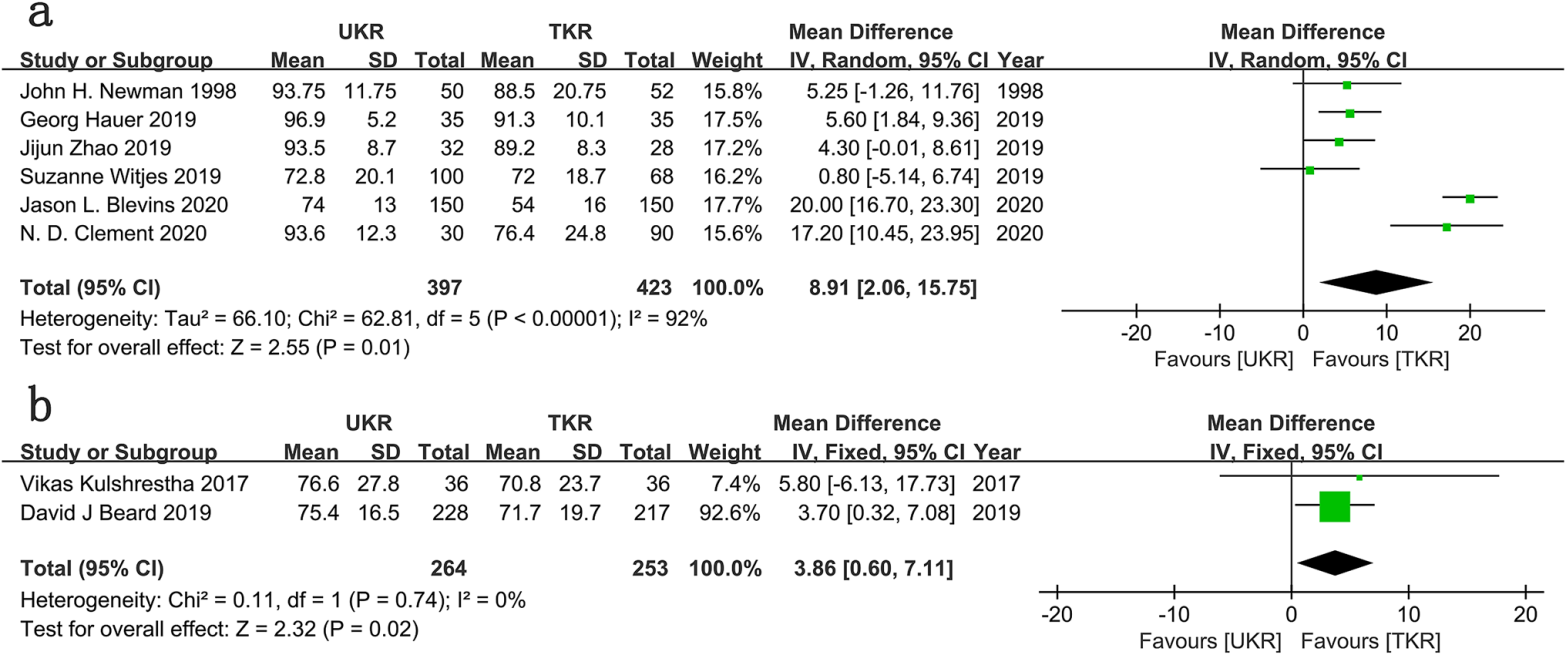
**a** Forest plots of the EQ-5D VAS scores of UKR and TKR; **b** Forest plots of the postoperative pain of UKR and TKR

Six studies involving 397 UKR patients and 423 TKR patients were included for the postoperative pain analysis. The pooled data demonstrated a significant benefit favoring UKR over TKR in pain score (WMD = 8.91; 95% CI = 2.06 to 15.75; *P* = 0.01; *I*^2^ = 92%; Fig. 6a). The source of the heterogeneity came from various pain scoring systems, including the Anterior Knee Pain Scale (AKPS), the Numeric Pain Rating Scale (NPRS), the Hospital for Special Surgery (HSS) pain score, and the visual analog scale of pain. The pooled data from two studies demonstrated a significant benefit favoring UKR over TKR in EQ-5D VAS score (WMD = 3.86; 95% CI = 0.60–7.11; *P* = 0.02; *I*^2^ = 0%; Fig. 6b).

#### Comparison of the postoperative complications

**Fig. 7:**
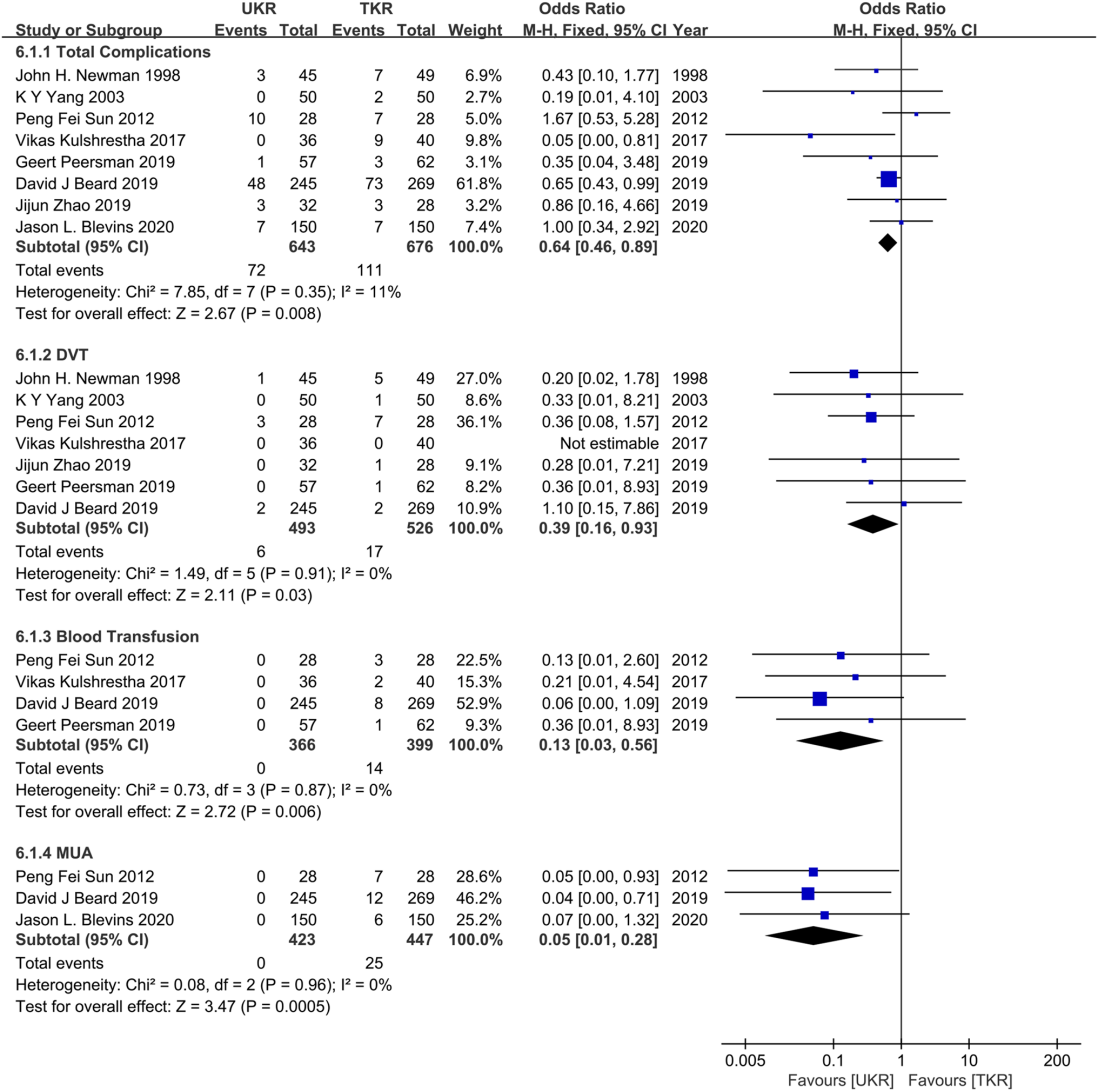
Forest plots of the complications of UKR and TKR

Total complications were counted in 8 studies involving 643 UKR patients and 676 TKR patients, deep vein thrombosis was counted in 7 studies, the blood transfusion rate was counted in 4 studies, and postoperative anesthesia reduction was counted in 3 studies. The pooled data demonstrated a significant benefit favoring UKR over TKR in total complications (OR = 0.64; 95% CI = 0.46–0.89; *P* = 0.008; *I*^2^ = 11%), DVT (OR = 0.39; 95% CI = 0.16–0.93; *P* = 0.03; *I*^2^ = 0%), blood transfusion rate (OR = 0.13; 95% CI = 0.03–0.56; *P* = 0.006; *I*^2^ = 0%), and MUA (OR = 0.05; 95% CI = 0.01–0.28; *P* = 0.0005; *I*^2^ = 0%) (Fig. 7).

#### Publication bias analysis

**Fig. 8:**
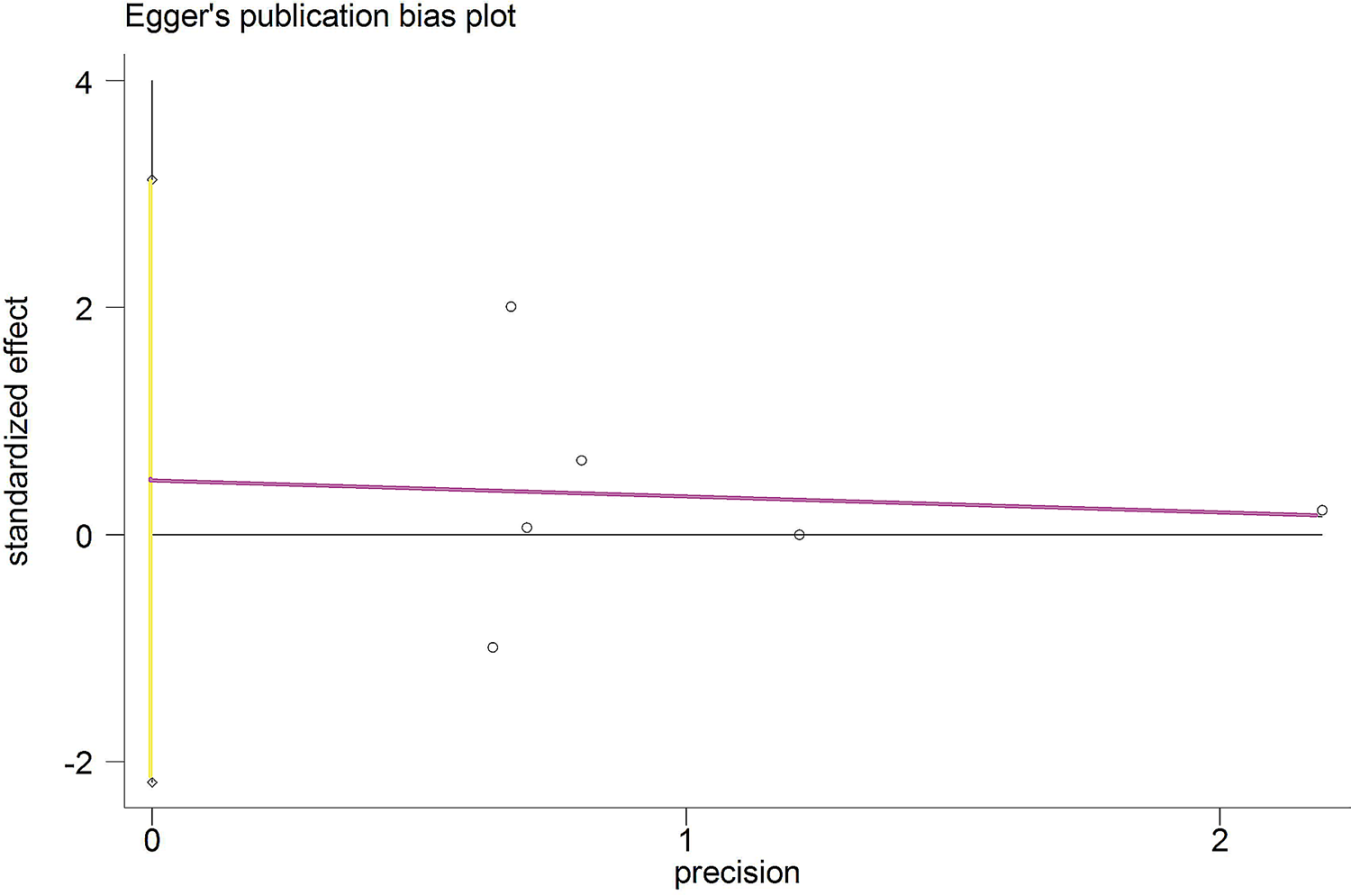
Egger’s publication bias plot for the revision

**Fig. 9:**
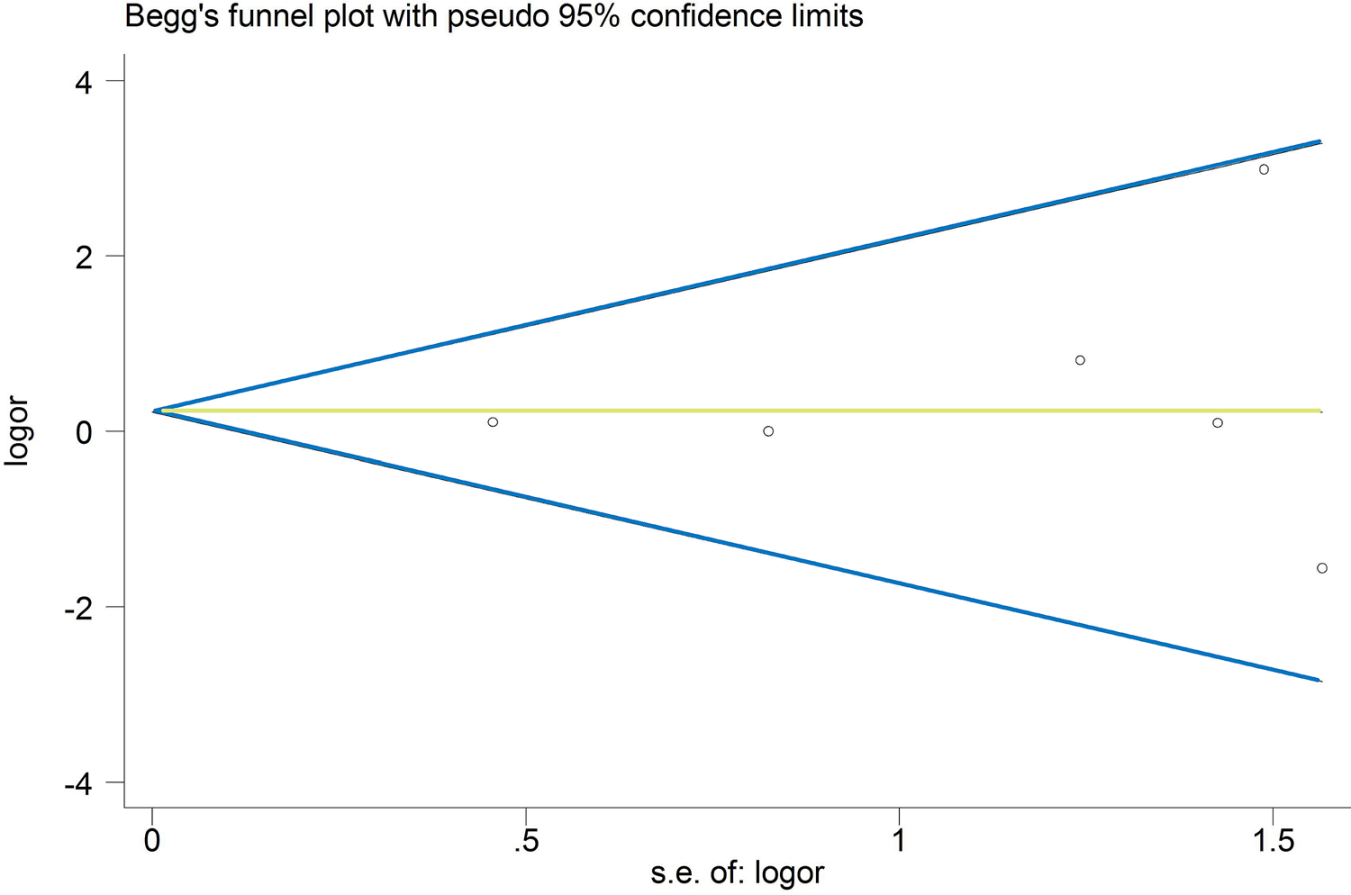
Funnel plot of Begg’s test for the revision publication bias

For the meta-analysis of UKR and TKR for revision, there were no evidence of publication bias via the formal statistical tests (Egger’s test, *P* = 0.648, Fig. 8; Begg’s test, *P* = 0.707, Fig. 9).

## Discussion

There is a debate in treatment choice and limited robust evidence to guide selection for the late-stage isolated medial knee OA. This study compared the patient-reported outcomes and complications of UKR and TKR in the early and middle follow-up.

The present meta-analysis showed that the patients in the UKR group gained better recovery than those in the TKR group including better postoperative knee general function and physical health, less pain and fewer complications within 5 years. These results suggested that UKR patients benefited more than TKR patients. This is probably ascribed to surgical characteristics of the UKR procedure that the surgeon only replaces the medial compartment of the knee by the prosthesis, protects the cruciate ligament and does not treat the other two chambers. This effectively reduces the influence on the biomechanics of the knee joint and enables patients undergoing UKR to recover faster and better [33–35]. Since Na et al. [36] claimed that the KSS did not distinguish between moderate and advanced functions of the knee well, the study contained HAAS to more accurately distinguish the differences. However, no significant differences were found in advanced knee function recovery. For OKS, UKR did not show superior outcomes among patients followed within 5 years. But constrained by the small number of studies, more studies are needed to verify this result of HAAS and OKS.

Compared to previous studies, there were no significant differences in postoperative revision between the two operations in the present study. Nearly all global registries [37, 38] (which comprise non-randomized, observational data) showed that the revision rate of UKR was higher than that of TKR, we think the following reasons could contribute to contradictory results. First, the UKR in previous studies could be constrained by techniques at that time, to a large extent, not as good as the present techniques. This could lead to the high revision rate of UKR since it is in consensus that surgical proficiency was inversely proportional to UKR revision rate in published studies [26, 39, 40]. The results in this study were obtained from the latest studies with the most up-to-date techniques and the impact of learning curves of UKR can be significantly minimized. As a consequence, the difference of postoperative revision of UKR and TKR was insignificant in the present study which probably could reflect the realistic situation of the two surgeries. Second, the higher rate of revision of UKR in published registries may be a result of a manifestation of inappropriate indications as well, which could not be avoided by surgical proficiency or robotic assistance [12]. Thirdly, the research subjects of these registries for the two operations might not be consistent. UKRs are generally performed in a younger, more active population group compared to TKR. High levels of activity increase the risk of implant loosening due to the production of wear which might lead to the high revision rate of UKR [25, 41]. Lastly, surgeons would probably modify UKR to TKR when unexplained pain occurs in patients done with UKR since the development of OA requires TKR as the final solution [42]. The revision of UKR under such cases reflect the routine treatment of OA rather than the UKR failure. In summary, this present study strictly stipulated the consistent inclusion exclusion criteria, so the results were more suitable for the comparison of the two operations for the simple medial knee OA.

Previous systematic review of four RCTs by Arirachakaran et al. [15] showed that UKR had no significant difference from TKR in knee function score and ROM and had fewer complications than TKR but a higher revision rate than TKR. However, two of their included studies involved patients having both surgeries on each of their knee, respectively, which may have had an impact on the accurate comparison of the results.

The following limitations of this meta-analysis should be acknowledged. 13 studies (RCTs and non-RCTs) were included due to limited literature available, and more RCTs are needed to obtain robust conclusions. Moreover, pre-caution should be taken to analyze the results of this study since the follow-up time for the studies included in this study varied from 0.5 to 5 years. Besides, substantial clinical heterogeneity could be introduced since studies were included from different countries where different diagnostic criteria for isolated medial knee OA might apply. Additionally, the detection bias existed inevitably as a result of the lack of the blindness in the evaluation of the results in three RCTs and self-reports of the evaluation indicators in non- RCTs.

## Conclusion

The past decade has seen an expanding interest in applying UKR for the treatment of isolated medial knee OA. However, arthroplasty surgeons preferred TKR due to the long learning curve and high revision of UKR from previous studies. Our meta-analysis of RCTs and non-RCTs shows that UKR effectively improves both health quality and knee function more than TKR in the early and mid-term follow-up. In addition, no significant differences were observed regarding postoperative revision. The reliable and robust evidence from this study is based on strict inclusion criteria. Future studies on more RCTs with less heterogeneity and less risk of bias, and longer follow-up reports are needed to fully evaluate the efficacy of the two operations for simple medial knee OA.

## Data Availability

All the data of the manuscript are presented in the paper.
